# Sevoflurane Inhibits Traumatic Brain Injury-Induced Neuron Apoptosis *via* EZH2-Downregulated KLF4/p38 Axis

**DOI:** 10.3389/fcell.2021.658720

**Published:** 2021-08-04

**Authors:** Zhongyu Wang, Juan Li, Anqi Wang, Zhaoyang Wang, Junmin Wang, Jingjing Yuan, Xin Wei, Fei Xing, Wei Zhang, Na Xing

**Affiliations:** ^1^Department of Anesthesiology and Perioperative Medicine, The First Affiliated Hospital of Zhengzhou University, Zhengzhou, China; ^2^Department of Human Anatomy, Basic Medical College of Zhengzhou University, Zhengzhou, China

**Keywords:** sevoflurane, traumatic brain injury, enhancer of zeste homolog 2, Krüppel-like factor 4, p38-mitogen-activated protein kinase pathway

## Abstract

Traumatic brain injury (TBI) is characterized by physical damage to the brain tissues, ensuing transitory or permanent neurological dysfunction featured with neuronal loss and subsequent brain damage. Sevoflurane, a widely used halogenated anesthetic in clinical settings, has been reported to alleviate neuron apoptosis in TBI. Nevertheless, the underlying mechanism behind this alleviation remains unknown, and thus was the focus of the current study. First, Feeney models were established to induce TBI in rats. Subsequently, evaluation of the modified neurological severity scores, measurement of brain water content, Nissl staining, and TUNEL assay were employed to investigate the neuroprotective effects of sevoflurane. Immunofluorescence and Western blot analysis were further applied to detect the expression patterns of apoptosis-related proteins as well as the activation of the p38-mitogen-activated protein kinase (MAPK) signaling pathway within the lesioned cortex. Additionally, a stretch injury model comprising cultured neurons was established, followed by neuron-specific enolase staining and Sholl analysis. Mechanistic analyses were performed using dual-luciferase reporter gene and chromatin immunoprecipitation assays. The results demonstrated sevoflurane treatment brought about a decrease blood-brain barrier (BBB) permeability, brain water content, brain injury and neuron apoptosis, to improve neurological function. The neuroprotective action of sevoflurane could be attenuated by inactivation of the p38-MAPK signaling pathway. Mechanistically, sevoflurane exerted an inhibitory effect on neuron apoptosis by up-regulating enhancer of zeste homolog 2 (EZH2), which targeted Krüppel-like factor 4 (KLF4) and inhibited KLF4 transcription. Collectively, our findings indicate that sevoflurane suppresses neuron apoptosis induced by TBI through activation of the p38-MAPK signaling pathway *via* the EZH2/KLF4 axis, providing a novel mechanistic explanation for neuroprotection of sevoflurane in TBI.

## Introduction

The last few years have witnessed the recognition of traumatic brain injury (TBI) as a risk factor for development of chronic traumatic encephalopathy, dementia and various neurodegenerative disorders, accompanied by brain structural and functional alternations (LoBue et al., 2019). Meanwhile, signaling molecules and metabolic derangements following TBI have been recognized to lead to blood-brain barrier (BBB) disruption, immune cell extravasation, and cerebral edema (Sulhan et al., 2020). Currently, available therapeutic modalities comprise of pharmacological intervention, biopharmaceuticals, and non-invasive interventions (brain stimulation and physical examination) (Pearn et al., 2017). However, patients who recover from TBI caused by an external force to the head often report experiencing further pain in a short/long term (Irvine and Clark, 2018), highly suggestive of the urgent need for more studies for TBI.

Sevoflurane, a volatile anesthetic with minimal effects on cerebral oxygen metabolism rate and intracranial pressure, is known to confer neuroprotective effects on the brain after ischemic insult by suppressing cell apoptosis (Wang et al., 2016; He et al., 2018). Meanwhile, sevoflurane post-conditioning was recently demonstrated to alleviate TBI-triggered neuronal apoptosis by promoting autophagy *via* the phosphatidylinositide 3-kinase/protein kinase B signaling pathway (He et al., 2018). More importantly, sevoflurane post-conditioning was reported to possess the ability to attenuate hypoxic-ischemic brain injury in neonatal rats by curbing excessive autophagy through up-regulation of enhancer of zeste homolog 2 (EZH2) (Xue et al., 2019). EZH2, a histone methyltransferase, can aggravate neurological deficits following ischemic stroke, while these deficits can be improved by EZH2 inhibitor DZNep (Chen et al., 2019). Furthermore, studies have documented that EZH2 can down-regulate Krüppel-like factor 4 (KLF4) by binding to its promoter (Shao et al., 2019). What’s more, Cui et al. (2017) reported that KLF4 knockdown can alleviate neuronal damage induced by TBI. Additionally, an abundance of signaling molecules downstream have been characterized for TBI by the hard-done work of our peers. Following ischemic injury, major mitogen-activated protein kinases (MAPKs) such as p38, and extracellular signal-regulated kinase (ERK) are known to play an important role in the mediation of neuronal death or survival (Zhang et al., 2015). In addition, delayed neuroprotection of sevoflurane was suggested to be partly dependent on p38-MAPK phosphorylation in rat models (Ye et al., 2012). In lieu of these findings, the current study set out to investigate the molecule mechanism underlying the neuroprotection of sevoflurane involving EZH2, KLF4, p38-MAPK signaling pathway both *in vivo* and *in vitro*.

## Materials and Methods

### Ethics Statement

Animal experimentation protocols in our study were approved by Animal Ethics Committee of The First Affiliated Hospital of Zhengzhou University. All animals were treated in accordance with the Guide for the Care and Use of Laboratory Animals published by the US National Institute of Health, and extensive efforts were made to minimize the suffering of the included animals.

### Model Establishment of Rats With TBI

In total, 66 specific-pathogen-free male Wistar rats (age: 2–3 months, weight: 190–220 g) were randomly assigned into 11 groups (*n* = 6), and individually housed in cages with free access to sterile food and drinking water under conditions including an artificial 12-h light/12-h dark cycle, with temperature conditions of 22–26°C in a 50% humidified environment.

Feeney’s method was employed to established rat models of TBI (He et al., 2018). First, the rats were intraperitoneally anesthetized with sodium pentobarbital (50 mg/kg), and then scalp was opened at 2 mm posterior to the right coronal suture and 2 mm from the mid-line. A 5 mm hole was drilled through the skull, and the dura mater was left intact. Subsequently, a 30 g hammer was dropped from a height of 20 cm to induce craniocerebral injury (impact force = 600 g/cm). The resultant bone hole was sealed with wax and the scalp was sutured. Rats in the sham group underwent the surgical procedure without the hammer drop.

Next, each rat was placed in an airtight anesthesia box (50 × 30 × 30 cm) with 1.5 cm diameter holes on two opposite sides for gas input 30 min and 60 min after the operation. The hole on one side was connected to a DragerFabius anesthesia machine (Drager, Germany) for input of sevoflurane with or without O_2_, while the hole on the other side was connected to a solar8000M Multifunction Monitor (GE, United States) for real-time concentration monitoring of sevoflurane, O_2_ and CO_2_. Rats were taken out of the anesthesia box after 60 min. The blood gas values of rats were within the normal range during inhalation.

Three rats died during TBI modeling, and were replaced by another three modeled rats. In total, 69 rats were used for experimentation, with a calculated death rate of approximately 4.3%. Subsequently, O_2_ (2 L/min) was delivered to sham-operated rats and rats with TBI. Simultaneously, O_2_ (2 L/min) containing 5% sevoflurane was delivered to the sham-operated rats, while O_2_ (2 L/min) containing 1, 3, and 5% sevoflurane was delivered to rats with TBI. After gas delivery, TBI rats were injected with 1% dimethyl sulfoxide (DMSO) or GSK126 (150 mg/kg) dissolved in 1% DMSO into the right ventricle following delivery of O_2_ (2 L/min) with or without 5% sevoflurane. Afterward, losmapimod (1.8 mg/kg) dissolved in 1% DMSO was intraperitoneally injected into the rats following delivery of O_2_ (2 L/min) with or without 5% sevoflurane (*n* = 11). Following treatment, the rats were placed back in the animal housing cages at constant temperature with free access to food. Except for nerve function assessment, tissue samples were collected from rats 3 days after modeling (He et al., 2018).

### Modified Neurological Severity Score (mNSS) Evaluation

The mNSS method was applied to evaluate the movement, feeling, reflex, muscle mass, abnormal behaviors, vision, sense of touch and balance of rats following different treatment protocols. mNSS was graded using a 0–10 point scale, wherein the maximum of mNSS value of 10 points was indicative of severe neurological deficit and 0 points suggested normal state. The detailed scoring criteria were as follows: presence of mono- or hemiparesis (1 point); inability to walk on a 3 cm wide beam (1 point); inability to walk on a 2 cm wide beam (1 point); inability to walk on a 1 cm wide beam (1 point); inability to balance on a 1 cm wide beam (1 point); inability to balance on a 0.5 cm diameter round stick (1 point); failure to exit a 30 cm diameter circle for 2 min (1 point); inability to walk a straight line (1 point); loss of startle behavior (1 point); loss of seeking behavior (1 point). Each point was awarded for failure to perform a task, such that higher scores indicated more severe neurological deficit. Neurological functions were evaluated on the 1st, 3rd, 7th, and 14th days after operation.

### BBB Permeability

Evans blue dye (0.2 mL/100 g) was injected through the femoral vein to determine the extent of TBI by examining the alterations in microvascular permeability. Following anesthesia induction with 1% pentobarbital sodium (30–40 mg/kg), the thoracic cavity was exposed, intracardiac perfusion was performed with heparin saline, and the brain tissues were weighed, and incubated with dimethylformamide for 60 h in a water bath at 60°C, and centrifuged at 1,000 × g for 5 min. Subsequently, the absorbance at a wavelength of 620 nm was measured using a spectrophotometer. Data analysis was performed using the Origin software (version s7.0), and Evans blue content was calculated from the previously plotted standard curve.

### Brain Water Content Determination

Rat brains were excised after euthanasia to evaluate neurological functions in different treatment groups. Cerebral cortex weighing 200 ± 20 mg was obtained at 2 mm from the edge of bone window. The blood and cerebrospinal fluid were removed using a filter paper. Brains were subsequently weighed [to measure the wet weight (WW)], dried at 70°C for 48 h, and weighed again [to measure the dry weight (DW)]. The brain water content was calculated as % water = (wet weight - dry weight)/wet weight × 100%.

### Nissl Staining

Brain tissues were treated with Nissl stain to analyze neurons. The cortical tissues surrounding the damaged area were collected, fixed with formaldehyde, embedded with paraffin, sliced into 4-μm sections, rinsed with distilled water, re-hydrated with alcohol of gradient concentrations, stained with Nissl (C0117, Beyotime, Shanghai, China) for 5 min and then rinsed with distilled water two times. Neurons in five randomly selected fields were microscopically observed to count the number of surviving neurons.

### Terminal-Deoxynucleotidyl Transferase-Mediated 2′-Deoxyuridine 5′-Triphosphate Nick-End Labeling (TUNEL) Staining

Tissue and cell apoptosis was detected using TUNEL kits (C1091, Beyotime) in strict accordance with the manufacture’s manuals. First, brain tissue sections were dewaxed with xylene, rinsed with alcohols of gradient concentrations, treated with DNase-free proteinase K at 37°C for 20 min, rinsed with phosphate buffered saline (PBS) thrice, and then incubated with 3% H_2_O_2_ for 20 min. Endogenous peroxidase was inactivated. Following incubation with TUNEL mixture for 1 h at 37°C, a HB050 inverted microscope (Zeiss, Hamburg, Germany) was introduced for imaging. The extent of brain injury was assessed using an apoptosis index, which was calculated as average percentage of TUNEL-positive cells in six fields of each section.

### Western Blot Analysis

Total protein content was extracted from cells or tissues using Radio Immunoprecipitation Assay lysis buffer containing phenylmethanesulfonyl fluoride (R0010, Solarbio, Beijing, China). The protein concentration was measured with a bicinchoninic acid kit (C503021-0500, Sangon Biotech, Shanghai, China). Next, the proteins were separated by sodium dodecylsulfate (SDS)-polyacrylamide gel electrophoresis and transferred onto a polyvinylidene fluoride membrane (Millipore, Billerica, MA, United States) using the wet-transfer method. The membrane was subsequently blocked in 5% bovine serum albumin (BSA) at room temperature for 1 h, incubated with diluted primary rabbit antibodies to Cleaved Caspase-3 (ab49822, dilution ratio of 1: 1,000, Abcam, Cambridge, United Kingdom), B-cell lymphoma-2 (Bcl-2) (ab182858, dilution ratio of 1: 2,000, Abcam), Bcl-2 associated protein X (Bax) (ab32503, dilution ratio of 1: 1,000, Abcam), EZH2 (ab191080, dilution ratio of 1: 2,000, Abcam), KLF4 (ab106629, dilution ratio of 1: 1,000, Abcam), H3K27me3 (ab6002, dilution ratio of 1: 1,000, Abcam), phosphorylated-p38 (p-p38) (ab47363, dilution ratio of 1: 1,000, Abcam), p-ERK1/2 (ab223500, dilution ratio of 1: 400, Abcam), glyceraldehyde-3-phosphate dehydrogenase (GAPDH) (ab8245, dilution ratio of 1: 5,000, Abcam), Hisyone H3 (ab1791, dilution ratio of 1: 1,000, Abcam) and β-actin (ab8226, dilution ratio of 1: 1,000, Abcam) at 4°C overnight. The membrane was rinsed with Tris Buffered saline Tween (TBST) for three times (15 min each) and incubated with horseradish peroxidase-conjugated goat anti-rabbit secondary antibody to immunoglobulin G (IgG) (ab6721, dilution ratio of 1: 5,000, Abcam) at room temperature for 1 h. After three TBST washes, the proteins were visualized and analyzed with the Bio-Rad gel imaging analysis system (Bio-Rad) and Image J software. The relative protein concentration was calculated as the ratio of gray value of protein to be tested to that of internal reference.

### Histoimmunochemistry

The paraffin-embedded sections were dewaxed and incubated with primary antibodies to EZH2 (ab186006, dilution ratio of 1: 1,000, Abcam), Cleaved Caspase3 (ab2302, dilution ratio of 1: 1,000, Abcam) and Bax (ab32503, dilution ratio of 1: 500, Abcam) and secondary antibody to IgG (ab150083, dilution ratio of 1: 100, Abcam). Non-specific normal IgG was employed as the negative control (NC). An optical microscope (XDS-800D, Caikon, Shanghai, China) was employed for observation and photography. The average absorbance of positive staining in each filed (200× or 400×) was calculated with the Image-Pro Plus 7.0 software, five fields in total.

### Reverse Transcription Quantitative Polymerase Chain Reaction (RT-qPCR)

Total RNA content was extracted from cells and tissues using TRIZOL kits (15596-018, Solarbio) to measure the purity and concentration of RNA. Subsequently, the obtained RNA was reverse transcribed into cDNA in accordance with the manual of PrimeScript RT reagent kit (RR047A, Takara, Japan). RT-qPCR was performed in YBR Green Master Mix (Life Technologies, Carlsbad, CA, United States) with the ABI PRISM 7500 RT-PCR system (Applied Biosystems, Foster City, CA, United States) for quantification on gene expression. All primer were designed and synthesized by Bioligo (Shanghai, China) as shown in Supplementary Table 1. The relative gene expression was analyzed using the 2^–ΔΔCt^ method with GAPDH serving as the internal control that △△Ct = (mean Ct value of target gene in the experimental group - mean Ct value of housekeeping gene in the experimental group) - (mean Ct value of target gene in the control group - mean Ct value of housekeeping gene in the control group).

### Bioinformatics Analysis

KLF4 gene sequences were analyzed with the help of the USCS database^1^. Downstream target genes of KLF4 were predicted by the hTFtarget database^2^. Kyoto Encyclopedia of Genes and Genomes (KEGG) pathway enrichment analysis was subsequently performed on the downstream target genes of KLF4 using the KOBAS3.0 database^3^.

### Isolation and Culture of Primary Cortical Neurons From Rats

Primary cortical neurons were isolated from embryos (developed for 17 days) of healthy, untreated and normally fed pregnant rats. Neuron medium was prepared by adding B27 (Invitrogen, Carlsbad, CA, United States) and penicillin-streptomycin (Invitrogen) into the neuron basic medium (Invitrogen). The cortex was isolated from rat embryos and cultured in the aforementioned medium to a concentration of 500,000 cells/mL. Next, 2 mL cell sample was implanted into the BioFlex 6-well plate coated with poly-L-ornithine-coated Silastic membranes (Flexcell International, Hillsborough, NC, United States) in a humidified incubator with 5% CO_2_ in air at 37°C. Half of the medium was renewed with fresh medium 2 days after culture and then renewed every 2 days. Neurons were cultured for 7 days in total *in vitro* prior to investigation.

### Model Establishment of Mechanical Neuronal Injury Induced by Stretching

As previously reported, cell injury controller II system (Custom Design & Fabrication, Richmond, VA, United States) was employed for mechanical neuronal injury model establishment. First, the cultured neurons were seeded in a BioFlex culture plate coated with polylysine for 3 days. The culture plate was connected with cell injury controller II system and neurons were stretched by 50 ms using positive pressure pulse of compressed nitrogen (peak pressure: 3.5–4.5 psi) to induce severe TBI. Every two wells without stretching were used as NCs in each plate. Finally, cell morphology was observed under an inverted microscope.

### Treatment of Neurons

Neurons were seeded in a 96-well plate at a density of 5 × 10^4^ cells/well in a humidified incubator at 37°C with 5% CO_2_ in air for 24 h. Next, the cells were placed in the aforementioned anesthesia box. The hole on one side was connected to a DragerFabius anesthesia machine (Drager) for gas input, while the hole on the other side was connected to a solar8000M Multifunction Monitor (GE) for real-time monitoring of the concentrations of sevoflurane, O_2_ and CO_2_. The gas containing 4% sevoflurane was input at 0.5 L/min for 10 min. Holes on both sides were closed. The sealed plexiglass box was treated for 3 h in a box at 37°C. The 96-well plate was then cultured in an incubator for 24 h, followed by cell proliferation and apoptosis detection using cell counting kit-8 (CCK-8) and flow cytometry, respectively.

Following sevoflurane treatment, cells were seeded in a 24-well plate at a density of 1.5 × 10^5^ cells/well and cultured for a period of 24 h. Next, short hairpin RNA (shRNA) against KLF4 (sh-KFL4) with multiplication of infection (MOI) being 100, 200, and 400, and corresponding NC (sh-NC) were delivered into the neurons. Then, the medium was renewed with complete medium for further culture 12 h later. Expression of green fluorescent protein (GFP) in sh-KLF4-treated neurons was observed under a fluorescent microscope. For verification purpose, KLF4 expression patterns were determined in sh-KLF4 or sh-NC-treated neurons by RT-qPCR after 3 days.

Grouping 1 included the control (normal neurons), stretch (stretched neurons), and stretch + Sevo (stretched neurons treated with 4% sevoflurane for 3 h) groups.

Grouping 2 included the stretch (stretched neurons), stretch + Sevo (stretched neurons treated with 4% sevoflurane for 3 h), stretch + Sevo + GSK126 + sh-NC (stretched neurons treated with 4% sevoflurane for 3 h, cultured in 10 μM GSK126 containing 1% DMSO and infected with sh-NC), stretch + Sevo + DMSO + sh-KLF4 (stretched neurons treated with 4% sevoflurane for 3 h, cultured in 1% DMSO and infected with sh-KLF4), and stretch + Sevo + GSK126 + sh-KLF4 (stretched neurons treated with 4% sevoflurane for 3 h, cultured with GSK126 dissolved in 1% DMSO and infected with sh-KLF4).

### Neuron-Specific Enolase (NSE) Staining and Sholl Analysis

Neuron purity was determined based on NSE positive results. In brief, immunofluorescence staining was performed using primary antibody of mouse anti-NSE (ab218388, dilution ratio of 1: 2,000, Abcam), followed by counterstaining with 4′,6-diamidino-2-phenylindole (DAPI) and F-actin dye liquor phalloidin. A FV1000 laser scanning cofocal microscope (Olympus, Tokyo, Japan) was then introduced for observation. Images were further analyzed by NeuronJ and Sholl analysis plug-in of the ImageJ software (Scion Corporation, Torrance, CA, United States). Sholl analysis was carried out as previously described (Langhammer et al., 2010) to quantify the number and length of neurons. The neuron length was estimated by measuring the distance from the cell body to the terminal of primary neurite (neurite directly originated from cell body).

### CCK-8

Neuron proliferation was detected strictly in accordance with the manual of the CCK-8 kit (C0037, Beyotime). First, the cells were incubated in a 96-well plate at 37°C following different treatment protocols. CCK-8 was added at 0, 24, 48, and 72 h for additional 1-h incubation. Next, cell proliferation was assessed by measuring the absorbance value at 450 nm using a microplate reader (Promega, Fitchburg, WI, United States).

### Flow Cytometry

Following different treatment protocols, neurons were collected and rinsed with PBS twice. Cell apoptosis was detected with reference to the manual of Annexin V-fluorescein isothiocyanate (FITC) apoptosis detection kit (Sigma-Aldrich, St. Louis, MO, United States). Cell density was adjusted to 3 × 10^5^ cells/mL using Binding Buffer. Subsequently, the cells were labeled with Annexin V-FITC and propidium iodide, followed by incubation at room temperature for 15 min. A flow cytometer (Becton Dickinson Labware, Franklin Lakes, NJ, United States) was then employed for cell apoptosis detection.

### Chromatin Immunoprecipitation (ChIP) Assay

Chromatin was isolated from cells, cross-linked with 1% formaldehyde and incubated with specific antibodies to EZH2 (ab228697, dilution ratio of 1: 200, Abcam) and H3K27me3 (ab6002, dilution ratio of 1: 200, Abcam). IP was performed using magnetic protein G immunomagnetic beads (Dynabeads, Life Technologies), followed by elution with an elution buffer solution (100 mM NaHCO_3_, and 1% SDS) at 65°C. Quantification was conducted using non-specific primers of binding sites to EZH2 on the KLF4 promoter by means of RT-qPCR. Rabbit IgG was used as NC for primary antibody.

### Dual-Luciferase Reporter Gene Assay

HEK-293T cells (ATCC, Manassas, VA, United States)^4^ were cultured in Dulbecco’s Modified Eagle Medium containing 10% fetal bovine serum in a humidified incubator at 37°C with 5% CO_2_ in air under saturated humidity.

The pGL3-KLF4 3′-untranslated region (3′-UTR) reporter vector was constructed by cloning sequences containing KLF4 promoter sites into the pGL3-Promoter luciferase reporter vector (Promega). Next, the pGL3-KLF4 3′-UTR reporter vector were co-transfected with oe-NC or oe-EZH2 into human HEK-293T cells using Lipofectamine 3000 (Invitrogen) for 48 h. The cell lysate was subsequently collected to measure the firefly and Renilla luciferase activities using a dual-luciferase reporter gene assay kit (Promega) with Renilla luciferase activity employed for standardization.

### Immunofluorescence

The rat cortical tissues were dewaxed with xylene, and hydrated with alcohols of gradient concentrations, followed by antigen retrieval overnight. Following incubation with 5% BSA for 1 h, the tissues were incubated with primary antibody to Ki67 (ab15580, dilution ratio of 1: 50, Abcam) at 4°C overnight and then incubated with fluorescence-supplemented secondary antibody of goat anti-rabbit IgG H&L (Alexa Fluor^®^488) (ab150081, dilution ratio of 1: 500, Abcam) at 37°C avoiding exposure to light. The excessive secondary antibody was rinsed off with PBS. Subsequently, the nuclei were stained with DAPI. Sections were then sealed with anti-quencher and observed under a fluorescence microscope (Leica Microsystems GmbH). By microscopic observation (200× or 400×), five fields were randomly selected to calculate the number of positively stained cells using the Image-Pro Plus 7.0 software. Ki67 positive rats = the number of Ki67-positive cells/the number of total cells × 100%.

### Statistical Analysis

Statistical analyses were performed using the SPSS 21.0 software (IBM, Armonk, NY, United States). Measurement data were expressed as mean ± standard deviation. Data comparison was analyzed by unpaired *t*-test between two groups, and comparisons among multiple groups were analyzed by one-way analysis of variance. Pairwise comparison within one group was analyzed by Tukey’s test. A value of *p* < 0.05 was regarded statistically significant.

## Results

### Sevoflurane Alleviated Neuron Apoptosis Induced by TBI

**FIGURE 1:**
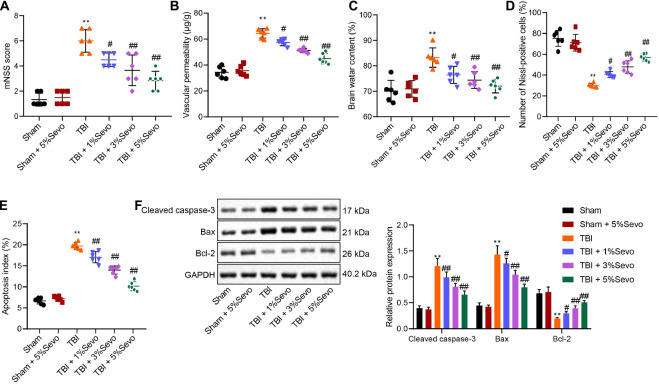
TBI-induced cell apoptosis was alleviated by sevoflurane 30 min after TBI modeling. **(A)** mNSS for evaluation on neurological functions of rats. **(B)** Vascular permeability of rats detected by Evans blue staining. **(C)** Brain water content. **(D)** Percentage of Nissl-positive cells. **(E)** Apoptosis index determined by TUNEL staining. **(F)** Protein levels of apoptosis-related factors (Cleaved Caspase-3, Bax and Bcl-2) in cortical tissues normalized to β-actin determined by Western blot analysis. **p < 0.001 vs. neurons from sham-operated rats. ^#^*p* < 0.05, ^##^*p* < 0.001 vs. neurons from rats with TBI. *n* = 6. The experiment was repeated three times independently.

Sevoflurane has been reported to alleviate TBI-induced cell apoptosis (He et al., 2018). To further verify this effect, we treated TBI rats with sevoflurane of different concentrations 30 min after TBI modeling. Neurological functions were subsequently evaluated by mNSS 3 days later, and it was found there were no significant differences before and after operation in sham-operated rats, whereas TBI rats presented with increased mNSS compared to sham-operated rats, yet mNSS decreased after the addition of sevoflurane (Figure 1A). Moreover, the reduction in mNSS was more profound with increases in sevoflurane concentration. Also, a similar changing tendency was observed in rats treated with sevoflurane 60 min after TBI modeling (Supplementary Figures 1, 3H). These findings indicated that sevoflurane was neuroprotective in rats with TBI to some degree.

Meanwhile, BBB is regarded as the specific structure in central nervous system that prevents macromolecular substances from entering brain parenchyma from peripheral blood for brain homeostasis maintenance. We injected differently treated mice with Evans blue dye liquor (0.2 mL/100 g) *via* femoral vein to detect BBB integrity. It was observed that BBB permeability was significantly increased in rats with TBI accompanied by damaged BBB, whereas these trends could be reversed by sevoflurane treatment (Figure 1B). Furthermore, elevated brain water content induced by TBI were noted to be diminished following sevoflurane treatment in a negative manner (Figure 1C).

Additionally, the percentage of Nissl-positive cells in rat brain tissues was detected to assess the morphological changes in cortical neurons. It was observed that sham-operated rats presented with clear and intact neurons without edema. Meanwhile, the neurons in rats with TBI were sparsely arranged without integrity, accompanied by cytoplasmic atrophy, oval or triangle nuclear and tumid cell body, indicative of typical neurodegeneration. Treatment with sevoflurane delayed the morphological changes in neurons to a certain extent. Compared with the sham-operated rats, reduced percentage of Nissl-positive cells was detected in rats with TBI, while sevoflurane treatment increased the percentage of Nissl-positive cells, and the increase was positively correlated with sevoflurane concentration (Figure 1D and Supplementary Figure 2A).

Furthermore, cortical neuron apoptosis was detected by TUNEL staining, which revealed that TBI rats exhibited high apoptosis rates, which were improved by sevoflurane of different concentrations (Figure 1E and Supplementary Figure 2B). Furthermore, levels of apoptosis-related factors (Cleaved Caspase-3, Bax, and Bcl-2) were quantified by Western blot analysis (Figure 1F). It was found that TBI rats presented with up-regulated Cleaved Caspase-3 and Bax levels along with down-regulated Bcl-2, whereas all these trends were reversed by sevoflurane treatment. Taken together, these results demonstrated that TBI-induced cell apoptosis was alleviated by sevoflurane.

### Sevoflurane Up-Regulates EZH2 to Inhibit TBI-Induced Neuron Apoptosis

**FIGURE 2:**
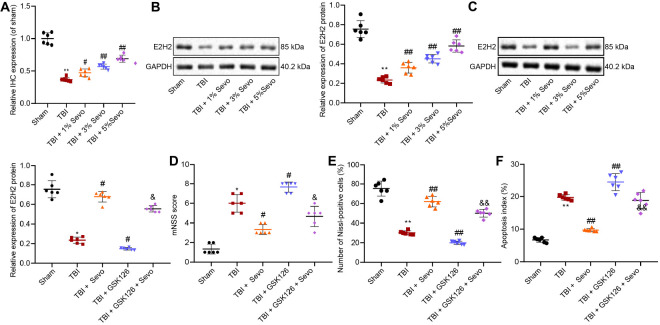
EZH2 downregulation aggravates TBI in contribution to neuron apoptosis. **(A)** Relative EZH2 expression in brain tissues treated with 1, 3, and 5% sevoflurane identified by immunohistochemistry. **(B)** EZH2 protein level in brain tissues treated with 1, 3, and 5% sevoflurane normalized to β-actin determined by Western blot analysis. **(C)** EZH2 protein level in brain tissues treated with 5% sevoflurane and GSK126 normalized to β-actin determined by Western blot analysis. **(D)** mNSS for evaluation on neurological functions of rats. **(E)** Percentage of Nissl positive cortical neurons. **(F)** Apoptosis index determined by TUNEL staining. Data were expressed as mean ± standard deviation. *p < 0.05 vs. neurons from sham-operated rats. ^#^*p* < 0.05 vs. neurons from rats with TBI. ^&^*p* < 0.05 vs. neurons from rats with TBI followed by treatment of 5% sevoflurane. *n* = 6. The experiment was repeated three times independently. ^#^*p* < 0.05, ^##^*p* < 0.01 vs. neurons from rats with TBI. ^&^*p* < 0.05, ^&&^*p* < 0.01 vs. neurons from rats with TBI followed by treatment of 5% sevoflurane.

Sevoflurane has been reported to confer a neuroprotective effect against hypoxic-ischemic brain damage by promoting the expression of EZH2 (Xue et al., 2019). To further explore the involvement of EZH2 in neuroprotection of sevoflurane, EZH2 expression patterns were determined in cortical tissues by means of immunohistochemistry (Figure 2A and Supplementary Figure 2C) and Western blot analysis (Figure 2B). The results demonstrated that EZH2 expression levels were reduced in TBI rats, and could be increased by sevoflurane in a positive manner, so 5% sevoflurane was selected for subsequent experiments. These results suggested that neuroprotection of sevoflurane in TBI might be associated with EZH2.

In order to further elucidate the role of EZH2 in TBI, we treated TBI rats with a selective EZH2 inhibitor, GSK126, followed by Western blot analysis (Figure 2C), mNSS (Figure 2D), Nissl staining (Figure 2E), and TUNEL staining (Figure 2F). The results demonstrated that in the presence of GSK126, EZH2 expression levels were decreased, mNSS was increased, percentage of Nissl-positive cells was reduced and cell apoptosis was promoted. On the other hand, decreased mNSS, increased percentage of Nissl-positive cells and suppressed cell apoptosis induced by sevoflurane were counterweighed by the addition of GSK126. These findings indicated that TBI alleviated by sevoflurane was aggravated by GSK126, an EZH2 inhibitor.

### Sevoflurane Inhibits Neuron Apoptosis by Promoting Target-Inhibition of EZH2 on KLF4 Transcription

**FIGURE 3:**
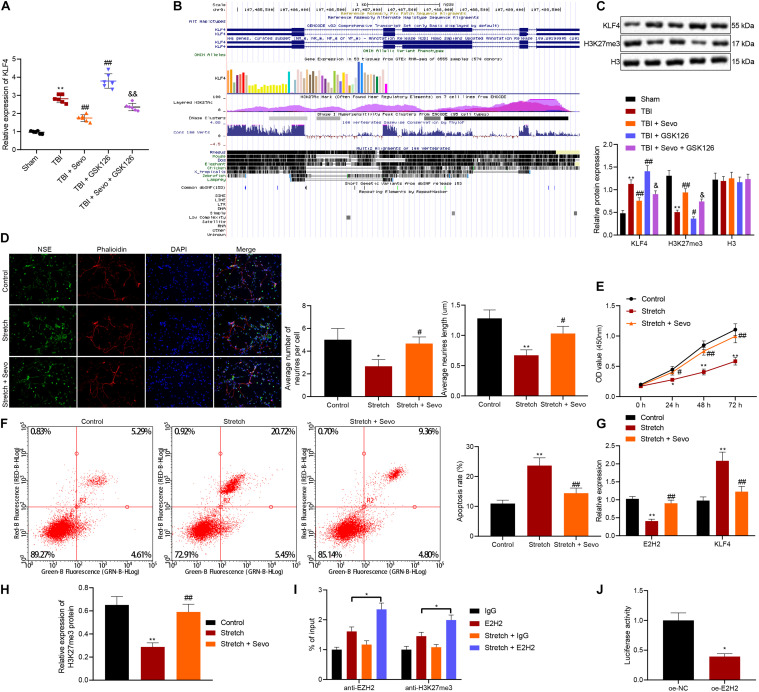
Promoted neuron apoptosis induced by stretching is curbed by sevoflurane through target-inhibition of EZH2 on H3K27 methylation in the KLF4 promoter region. **(A)** KLF4 mRNA expression in rat brain tissues determined by RT-qPCR. *n* = 6. *p < 0.05 vs. neurons from sham-operated rats. ^#^*p* < 0.05 vs. neurons from rats with TBI. ^&^*p* < 0.05 vs. neurons from rats with TBI followed by treatment of 5% sevoflurane. **(B)** KLF4 sequence analysis, including H3K27 methylation in the KLF4 promoter region. **(C)** Protein levels of KLF4 and H3K27me3 in rat brain tissues determined by Western blot analysis. **(D)** Neuronal structure following treatment of stretching and 4% sevoflurane. Yellow arrows indicate reduced neurite and formation of neuronal network. Green: NSE staining. Blue: DAPI-stained nuclei. Red: cytoskeleton stained by rhodamine phalloidin. **(E)** Cortical neuron proliferation detected by CCK-8 assay. **(F)** Cortical neuron apoptosis detected by flow cytometric analysis. **(G)** mRNA expression of EZH2 and KLF4 determined by RT-qPCR. **(H)** H3K27me3 protein level determined by Western blot analysis. **(I)** Expression of EZH2 and H3K27me3 in the KLF4 promoter region in stretched neurons analyzed by ChIP-qPCR. *p < 0.05. **(J)** KLF4 promoter activity affected by EZH2 in 293T cells through dual-luciferase reporter gene assay. *p < 0.05 vs. 293T cells treated with oe-NC. Data were expressed as mean ± standard deviation. *p < 0.05 vs. normal neurons. ^#^*p* < 0.05 vs. stretched neurons. The experiment was repeated three times independently. **p < 0.01 vs. neurons from sham-operated rats. ^##^*p* < 0.01 vs. neurons from rats with TBI. ^&&^*p* < 0.01 vs. neurons from rats with TBI followed by treatment of 5% sevoflurane.

Recent studies suggest that KLF4 promotes TBI-induced nerve injury (Cui et al., 2017). RT-qPCR was subsequently performed to determine KLF4 mRNA expression patterns in rat brain tissues (Figure 3A), which revealed that KLF4 levels were up-regulated in rats with TBI, while being down-regulated by additional treatment of sevoflurane, the action of which was reversed by the addition of GSK126. Moreover, combined treatment of sevoflurane and GSK126 induced prominent up-regulation of KLF4 compared to individual treatment with sevoflurane. These results elucidated that neuroprotection of EZH2 in TBI might depend on KLF4 down-regulation.

Furthermore, EZH2 has been documented to inhibit KLF4 expression by binding to KLF4 promoter (Shao et al., 2019). Prediction results from UCSC database suggested the presence of H3K27 methylation sites in the KLF4 promoter region (Figure 3B). To further verify the relation between EZH2 and KLF4, the expression patterns of KLF4 and H3K27me3 in brain tissues were determined by Western blot analysis (Figure 3C), and the results demonstrated that TBI rats presented with high KLF4 expression and low H3K27me3 expression levels, both of which were reversed by sevoflurane treatment. It was further observed that the action of sevoflurane was further restored by the addition of GSK126. Meanwhile, when sevoflurane and GSK126 were co-present, KLF4 expression levels were significantly increased and those of H3K27me3 were decreased compared to individual treatment with sevoflurane. These findings indicated that EZH2 might down-regulate KLF4 expression by promoting H3K27 methylation on the KLF4 promoter region.

To further investigate the relation between EZH2 and KLF4, rat neurons were cultured, induced with mechanical injury, and treated with 4% sevoflurane for 2 h, followed by another 24-h of culture. Morphology of cortical neurons was subsequently observed under a microscope (Figure 3D), and it was found that unstretched neurons were featured with large nuclei, oval cell body and lots of smooth multipolar neurite. In addition, neurons were injured, cell body was shrunk and neurite was reduced 24 h after stretching. Further delivery of sevoflurane decreased cell body shrinkage, increased multipolar neurite and improved neuron phenotypes. Neurite growth is regarded an important morphological characteristic of neurons. Sholl analysis was then performed to calculate the number and length of neurites, and the results showed that the number of neurites exposed to stretching was smaller, and average length was reduced, both of which were reversed by sevoflurane treatment. CCK-8 (Figure 3E) and flow cytometric analyses (Figure 3F) revealed that treatment with sevoflurane suppressed cell proliferation and facilitated cell apoptosis induced by stretching.

Furthermore, RT-qPCR (Figure 3G) and Western blot analysis (Figure 3H) were performed for quantifying the expression patterns of EZH2, KLF4, and H3K27me3, which revealed that down-regulation of EZH2, and H3K27me3 along with up-regulation of KLF4 triggered by stretching were counteracted by sevoflurane.

Additionally, ChIP-qPCR was applied to analyze the enrichment of EZH2 and H3K27me3 in the KLF4 promoter region in stretched neurons to further explore the targeting relation between EZH2 and KLF4 (Figure 3I). In addition, the addition of sevoflurane enriched more EZH2 and H3K27me3 in the KLF4 promoter region. The targeting relation between EZH2 and KLF4 3′-UTR was confirmed by dual-luciferase reporter gene assay (Figure 3J), as evidenced by over-expressed EZH2 corresponded to reduced relative luciferase activity, indicating that over-expression of EZH2 decreased the activity of KLF4 promoter and EZH2 inhibited KLF4 transcription.

### Sevoflurane Alleviates TBI-Induced Neuron Apoptosis by Inhibiting KLF4 *via* EZH2

**FIGURE 4:**
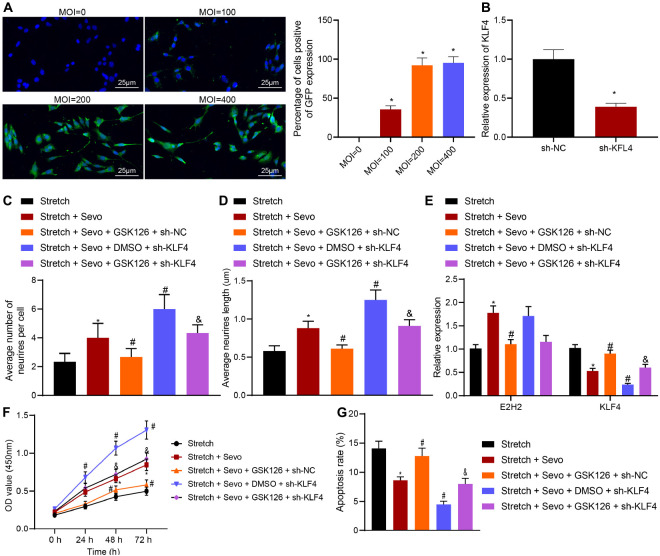
TBI-induced neuron apoptosis is alleviated by sevoflurane via EZH2/KLF4. **(A)** MOI value of sh-KLF4 in cortical neurons detected by immunohistochemistry. **(B)** Infection efficiency of sh-KLF4 in cortical neurons determined by RT-qPCR. **(C)** Average number of neurite in stretched cortical neurons treated with 4% sevoflurane with or without GSK126/sh-KLF4 analyzed by Sholl. **(D)** Average neurite length in stretched cortical neurons treated with 4% sevoflurane with or without GSK126/sh-KLF4 analyzed by Sholl. **(E)** mRNA expression of EZH2 and KLF4 in neurons determined by RT-qPCR. **(F)** Neuron proliferation detected by CCK-8 assay. **(G)** Neuron apoptosis detected by flow cytometry. Data were expressed as mean ± standard deviation. *p < 0.05 vs. stretched neurons. ^#^*p* < 0.05 vs. stretched neurons treated with 4% sevoflurane. ^&^Stretched neurons treated with 4% sevoflurane, GSK126 and sh-NC. The experiment was repeated three times independently.

To further verify that EZH2 mediates neuron apoptosis by regulating KLF4 expression after sevoflurane treatment, EZH2 inhibitor GSK126 and/or sh-KLF4 were delivered into cortical neurons. Subsequent observation under a fluorescence microscope illustrated that virus-infected neurons exhibited green fluorescent nucleus and cytoplasm with uneven fluorescence intensity. When MOI = 200, there were more than 90% green fluorescent cells positive for GPF. Meanwhile, RT-qPCR results for determination of KLF4 expression patterns in sh-KLF4 or sh-NC-treated neurons on the 3rd day after infection demonstrated that KLF4 expression levels were significantly reduced in the presence of sh-KLF4 (*p* < 0.05), indicating successful construction of neurons with silenced KLF4 (Figures 4A,B).

Additional results of Sholl analysis demonstrated that the presence of GSK126 and sevoflurane resulted in fewer neurite and shorter neurite length. Meanwhile, the number and length of neurites were found to be increased by sh-KLF4 treatment compared to individual sevoflurane treatment. In addition, GSK126-induced decreased number and length of neurite were increased by combined treatment with GSK126 and sh-KLF4 (Figures 4C,D).

Further results of RT-qPCR showed that addition of GSK126 decreased the up-regulation of EZH2 induced by sevoflurane and increased the expression of KLF4. In stretched neurons treated with sevoflurane following delivery of sh-KLF4, KLF4 expression levels were observed to be significantly reduced, while those of EZH2 were unaltered. Moreover, combined treatment with sh-KLF4 and GSK126 in the presence of sevoflurane down-regulated the KLF4 expression, but did not alter the EZH2 expression (Figure 4E). Subsequent findings from CCK-8 assay and flow cytometry revealed that GSK126 suppressed the enhanced cell proliferation and promoted the inhibited cell apoptosis induced by sevoflurane while knocking down KLF4, contributing to cell proliferation and curbed cell apoptosis. Furthermore, additional KLF4 knockdown in the presence of GSK126 gave an impetus to cell proliferation and caused the inhibition of cell apoptosis (Figures 4F,G). Taken together, these results indicated that sevoflurane up-regulated EZH2 to inhibit KLF4, by which neuron apoptosis was suppressed in TBI.

### EZH2/KLF4 Axis Inactivates the p38-MAPK Signaling Pathway to Inhibit TBI-Induced Cell Apoptosis

**FIGURE 5:**
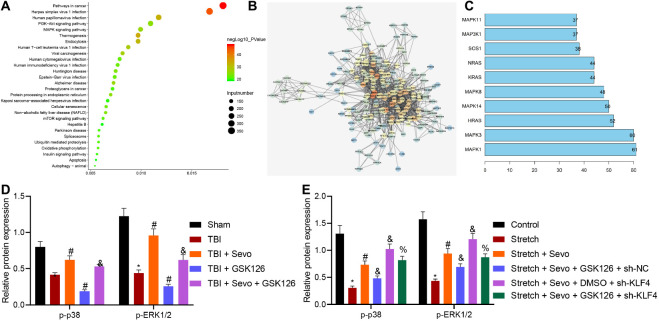
EZH2 inactivates the p38-MAPK signaling pathway by mediating KLF4 to inhibit p38 phosphorylation. **(A)** KEGG pathway enrichment analysis of KLF4 downstream target genes. The abscissa refers to GeneRatio, the ordinate refers to KEGG items, the size of circles refers to the number of genes enriched in items, color indicates enriched *p-*values and the right histogram indicates color scale. **(B)** Interaction analysis of candidate genes enriched in the MAPK signaling pathway. Each circle indicates each gene, and the line between genes indicates an interaction. Darker color suggests core position and higher degree values correspond to more interacted genes. **(C)** Degree values of core genes in the gene interaction network. The abscissa refers to degree values, and the ordinate refers to gene names. **(D)** Protein levels of p38 signaling pathway-related proteins in stretched cortical neurons treated with 3% sevoflurane with or without EZH2 inhibitor GSK126 (150 mg/kg) and/or sh-KLF4 determined by Western blot analysis. **(E)** Protein levels of p38 signaling pathway-related proteins in rat brain tissues treated with 5% sevoflurane or GSK126 (150 mg/kg) determined by Western blot analysis. Data were expressed as mean ± standard deviation. *n* = 6. *p < 0.05 vs. sham-operated rats or normal neurons. ^#^*p* < 0.05 vs. rats with TBI or stretched neurons. ^&^Stretched neurons treated with sevoflurane. ^%^*p* < 0.05 vs. stretched neurons treated with sevoflurane, GSK126, and sh-NC. The experiment was repeated three times independently.

To further understand the downstream regulatory mechanism of KLF4, downstream target genes of KLF4 were predicted, followed by KEGG pathway enrichment analysis (Figure 5A). It was found that target genes of KLF4 were primarily enriched in the MAPK signaling pathway. Meanwhile, the p38 (MAPK14) signaling pathway is known to inhibit TBI-induced cell apoptosis (Shi et al., 2018). Candidate genes of the MAPK signaling pathway were subsequently selected from KEGG enrichment results of KLF4 target genes, followed by gene interaction analysis to establish a gene interaction network. The degree values of core genes showed that MAPK14 (p38) was localized at the core of gene interaction network (Figures 5B,C), suggesting that KLF4 might mediate TBI-induced cell apoptosis through the p38-MAPK signaling pathway.

Additionally, the expression patterns of p38-MAPK signaling pathway-related protein levels were determined *in vivo*, and the results demonstrated reduced phosphorylation of p38 and ERK1/2 and inactivated p38-MAPK signaling pathway in brain tissues of rats with TBI. Opposite results were observed in the presence of sevoflurane, while similar trends were detected upon the addition of GSK126. Moreover, co-presence of sevoflurane and GSK126 resulted in reduced phosphorylation of p38 and ERK1/2 and inactivated p38-MAPK signaling pathway than sevoflurane presence alone. These results suggested that the inhibitory action of sevoflurane on neuron apoptosis in TBI was associated with activation of the p38-MAPK signaling pathway (Figure 5D and Supplementary Figure 3A).

Additional *in vitro* determination on p38-MAPK signaling pathway-related protein levels revealed reduced phosphorylation of p38 and ERK1/2 in stretched cortical neurons. Meanwhile, opposite results were observed in the presence of sevoflurane, action of which was abrogated following the addition of GSK126. Further delivery of sh-KLF4 increased the phosphorylation of p38 and ERK1/2 (Figure 5E and Supplementary Figure 3B). Collectively, these results demonstrated that sevoflurane activated the p38-MAPK signaling pathway, which was inactivated when EZH2 was inhibited. Inhibitory effect of GSK126 on the p38-MAPK signaling pathway was reversed by silencing KLF4, indicating that KLF4 mediated cell apoptosis induced by TBI *via* the p38-MAPK signaling pathway.

### Sevoflurane Suppresses Neuron Apoptosis Induced by TBI by Mediating EZH2 Expression *via* the KLF4/p38 Signaling Pathway

**FIGURE 6:**
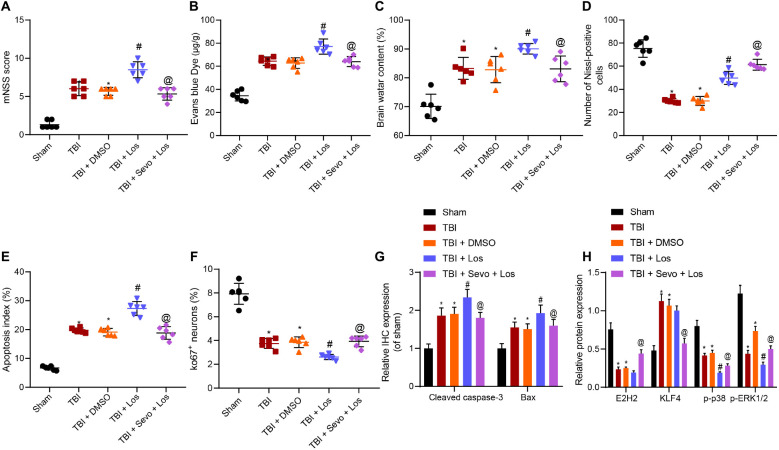
Inhibitory action of sevoflurane on neuron apoptosis in rats with TBI depends on the EZH2/KLF4/p38 axis. **(A)** mNSS for evaluation on neurological functions. **(B)** Evans blue (0.2 mL/100 g) staining results. **(C)** Brain water content. **(D)** Number of Nissl-positive cells. **(E)** Neuron apoptosis index detected by TUNEL staining. **(F)** Ki67 expression in rat brain tissues identified by immunohistochemistry. **(G)** Expression of Cleaved caspase3 and Bax identified by immunohistochemistry. **(H)** Expression of EZH2, KLF4, p-p38, and p-ERK1/2 in rat brain tissues determined by Western blot analysis. Data were expressed as mean ± standard deviation. *n* = 6. *p < 0.05 vs. sham-operated rats. ^#^*p* < 0.05 vs. rats with TBI. ^@^*p* < 0.05 vs. TBI rats treated with losmapimod. The experiment was repeated three times independently.

A rat model of TBI was established to verify that sevoflurane inhibits TBI-induced neuron apoptosis by promoting the activation of the p38 signaling pathway through KLF4 downregulation *via* EZH2 *in vivo*. The p38 signaling pathway was inactivated using p38 MAPKα/β inhibitor losmapimod, and TBI rats were treated with losmapimod and sevoflurane. Neurological functions were evaluated by mNSS 3 days later (Figure 6A), followed by BBB detection (Figure 6B) and brain water content determination (Figure 6C). The obtained results demonstrated that losmapimod led to higher mNSS, increased BBB permeability and increased brain water content, all of which were reversed by treatment with sevoflurane.

In addition, the percentage of Nissl-positive cells in rat brain tissues and neuron apoptosis were determined by Nissl staining (Figure 6D and Supplementary Figure 3C) and TUNEL staining (Figure 6E and Supplementary Figure 3D), respectively. The results of which showed that losmapimod elevated the TBI-induced decreased percentage of Nissl-positive cells and promoted neuron apoptosis, both of which were reversed by additional sevoflurane treatment.

Immunohistochemistry was subsequently employed to determine the expression patterns of Ki67, Cleaved Caspase-3 and Bax (Figures 6F,G and Supplementary Figures 3E,F). It was found that TBI decreased the Ki67 expression, while losmapimod treatment resulted in down-regulated Ki67 and increased expression levels of Cleaved Caspase-3 and Bax induced by trauma. Further delivery of sevoflurane increased the Ki67 expression while reducing those of Cleaved Caspase-3 and Bax, indicated that losmapimod suppressed cortical tissue proliferation by inhibiting the p38 signaling pathway, the action of which can be counterweighed by sevoflurane.

Western blot analysis for quantification of EZH2, KLF4, and p38 pathway-related proteins revealed no significantly differed expression of EZH2 and KLF4 affected by losmapimod, which reduced the extent of p38 and ERK1/2 phosphorylation (Figure 6H and Supplementary Figure 3G). When sevoflurane was supplemented, EZH2 was found to be significantly up-regulated, KLF4 expression levels were decreased and the extent of p38 and ERK1/2 phosphorylation was elevated.

Taken together, these results indicated that losmapimod inhibited the p38 signaling pathway to suppress cortical tissue proliferation, which was neutralized by treatment with sevoflurane.

## Discussion

TBI, caused by a blow, bump or jolt to the head, results in brain dysfunction yet no officially approved therapeutic modalities are currently available for TBI (Wang et al., 2018; Capizzi et al., 2020). Sevoflurane post-conditioning has been indicated to protect against brain injury induced by middle cerebral artery occlusion in rat models by inhibiting autophagy and apoptosis (Shi et al., 2020), highly suggestive of its neuroprotective ability. In an effort to expand our understanding, the current study set out to uncover the mechanism underlying the functional role of sevoflurane in neuron apoptosis induced by TBI. Collectively, our findings demonstrated that sevoflurane attenuated TBI-triggered neuron apoptosis by activating the p38-MAPK signaling pathway through inhibition of KLF4 *via* EZH2 up-regulation.

One of the most instrumental findings in our study was the neuroprotective effect of sevoflurane against TBI by curbing neuron apoptosis, as evidenced by down-regulated expressions of Bax (pro-apoptotic protein) and Cleaved Caspase-3 and up-regulated Bcl-2 levels (anti-apoptotic protein). Similarly, administration of sevoflurane has been previously documented to augment Bcl-2 protein levels and decrease those of Cleaved Caspase-3 in rat brain cerebral ischemia-reperfusion models, a reflection of diminished neuron apoptosis (Shi et al., 2020). In addition, another study highlighted that sevoflurane post-conditioning conferred an inhibitory effect on cell apoptosis in cerebral ischemia-reperfusion injury, which is largely in agreement with our findings (Kim et al., 2017). Meanwhile, sevoflurane post-conditioning is also known to up-regulate EZH2 levels in hypoxic-ischemic brain injury (Xue et al., 2019). Consistently, our findings demonstrated that despite being poorly expressed in cortical tissues of rats, EZH2 exhibited a significant increase as a result of sevoflurane treatment. More importantly, existing evidence reflects that ischemic/reperfusion injury can induce high levels of EZH2 in microglia, while on the other hand, inhibition of EZH2 attenuates brain injury in mice triggered by middle cerebral artery occlusion (Chen et al., 2019). Furthermore, EZH2 inhibition can further ameliorate neuroinflammation following subarachnoid hemorrhage in rats, which underscores the neuroprotective function of EZH2 inhibition (Luo et al., 2020). These results suggest that sevoflurane and EZH2 levels play a deciding role in the fate of TBI.

Additional experimentation in our study suggested that components of the EZH2-mediated KLF4/p38/MAPK signaling pathway were under the mediation of sevoflurane during the inhibition of neuron apoptosis, such that sevoflurane promotes the EZH2 expression to inhibit KLF4 transcription, consequently inactivating the p38-MAPK signaling pathway. The study performed by Shao et al. (2019) uncovered similar results, wherein deficiency of EZH2 could promote the transcription of KLF4. Moreover, down-regulation of KLF4 was previously noted in neural stem cells for mediation of axonal regeneration, and further highlighted to be of great importance to neural development, while dysregulation of KLF4 is known to precipitate hydrocephalus (Qin et al., 2011; Qin and Zhang, 2012). Further in agreement with our results, another study indicated that KLF4 expression levels could be augmented by sevoflurane (Yamamoto et al., 2020). Furthermore, KLF4 has been shown to activate the p38-MAPK signaling pathway in osteosarcoma cancer stem cells (Qi et al., 2019). On the other hand, Shi et al. (2018) demonstrated that resveratrol can improve cognitive deficits following TBI by activating the p38 signaling pathway. Besides, up-regulation of p38-MAPK was previously implicated in preventing cerebrovascular autoregulation impairment following TBI (Armstead et al., 2013). It is noteworthy that, p-p38 has been observed to be up-regulated in a time-dependent manner following treatment with 3% sevoflurane in hippocampal neurons, which are damaged by sevoflurane (Song et al., 2020). Together, these findings and data indicate that appropriate concentration and period of sevoflurane treatment warrants further exploration in the future for clinical application.

## Conclusion

In summary, findings uncovered in our study demonstrate the neuroprotective effect of sevoflurane in TBI depended on EZH2 and KLF4 as downstream molecules and the p38-MAPK signaling pathway, such that the reported axis reduces neuron apoptosis following TBI. Our novel findings provide a theoretic basis for developing clinically effective therapeutic strategies for TBI. Nevertheless, we cannot exclude the involvement of other signaling pathways in the neuroprotective action of sevoflurane due to the complex microenvironments and the interaction of p38-MAPK pathways with other signaling pathways. In addition, concentration of sevoflurane should be determined in case of any side effects in future investigations.

## Data Availability Statement

The original contributions presented in the study are included in the article/Supplementary Material, further inquiries can be directed to the corresponding author/s.

## Ethics Statement

The animal study was reviewed and approved by the Animal Ethics Committee of the First Affiliated Hospital of Zhengzhou University.

## Author Contributions

ZhoW, JL, AW, and ZhaW designed the study. JW, JY, XW, and FX collated the data, carried out data analyses, and produced the initial draft of the manuscript. ZhoW, JL, WZ, and NX contributed to drafting the manuscript. All authors have read and approved the final submitted manuscript.

## Conflict of Interest

The authors declare that the research was conducted in the absence of any commercial or financial relationships that could be construed as a potential conflict of interest.

## Publisher’s Note

All claims expressed in this article are solely those of the authors and do not necessarily represent those of their affiliated organizations, or those of the publisher, the editors and the reviewers. Any product that may be evaluated in this article, or claim that may be made by its manufacturer, is not guaranteed or endorsed by the publisher.

## Footnotes

1
http://genome.ucsc.edu/

2
http://bioinfo.life.hust.edu.cn/hTFtarget#!/

3
http://kobas.cbi.pku.edu.cn/kobas3/?t=1

4
http://www.bnbio.com/pub/search.asp?Keyword=HEK-293T
