# Supplementary material for: Sevoflurane Inhibits Traumatic Brain Injury-Induced Neuron Apoptosis via EZH2-Downregulated KLF4/p38 Axis
Source: Front Cell Dev Biol. 2021 Aug 4;9:658720. doi: 10.3389/fcell.2021.658720 (PMC8371463; doi:10.3389/fcell.2021.658720)
Supplement: Supplementary file 1 [file Data_Sheet_1.docx]

**
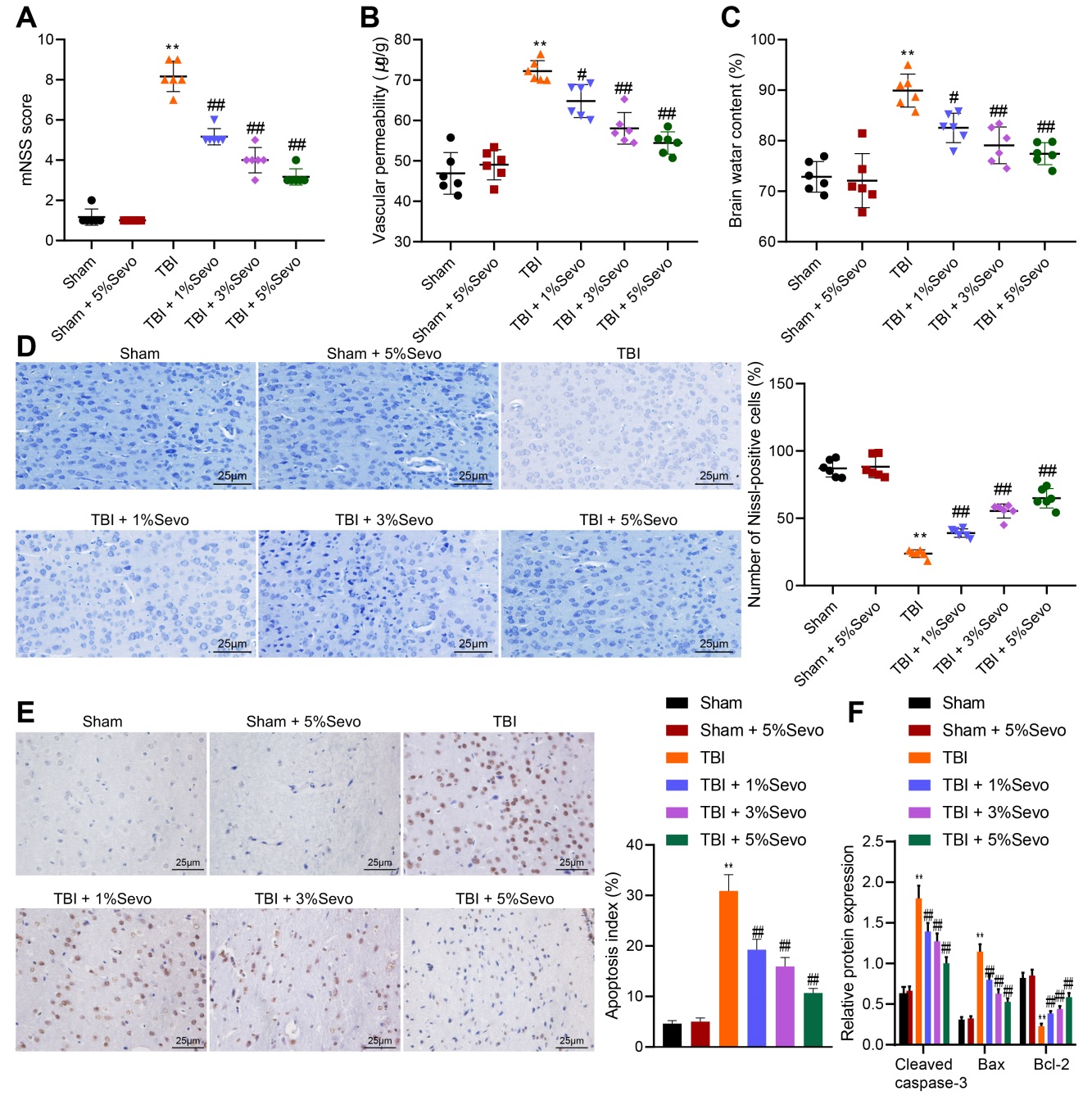
**

**FIGURE S1 | TBI-induced cell apoptosis was alleviated by sevoflurane 60 min after TBI modeling.** A, mNSS for evaluation on neurological functions of rats. B, Vascular permeability of rats detected by Evans blue staining. C, Brain water content. D, Percentage of Nissl-positive cells. E, Apoptosis index determined by TUNEL staining. F, Protein levels of apoptosis-related factors (Cleaved caspase-3, Bax and Bcl-2) in cortical tissues normalized to β-actin determined by Western blot analysis. * *p* < 0.05, ** *p* < 0.001 *vs.* neurons from sham-operated rats. # *p* < 0.05, ## *p* < 0.001 *vs.* neurons from rats with TBI. n = 6. The experiment was repeated 3 times independently.

**
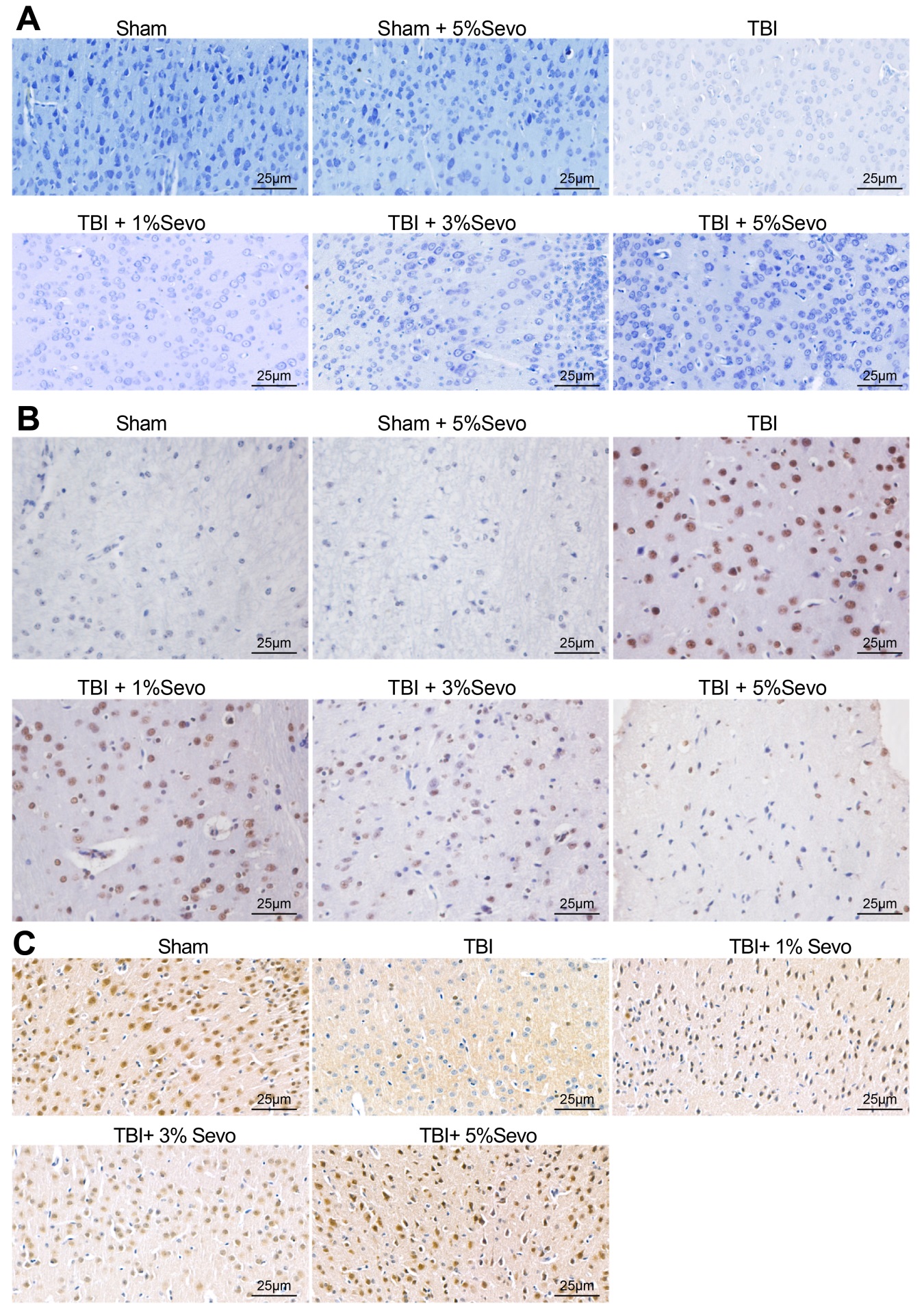
**

**FIGURE S2 | Representative experimental images.** A, Representative images of Nissl staining for Figure 1D; B, Representative images of TUNEL staining for Figure 1E; C, Representative images of immunohistochemistry for Figure 2A.

**
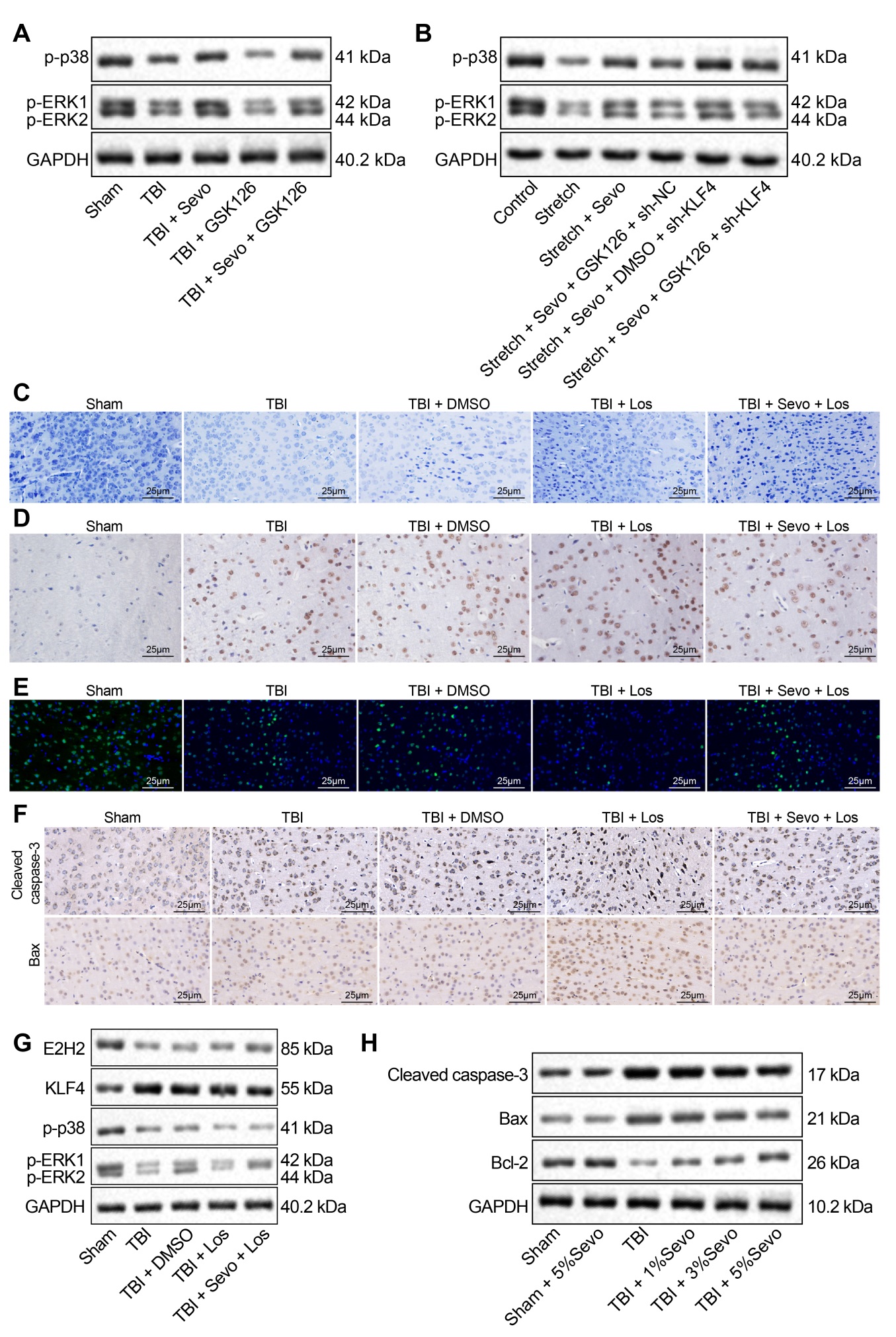
**

**FIGURE S3 | Representative experimental images.** A, Protein blots for Figure 5D; B, Protein blots for Figure 5E; C, Representative images of Nissl staining for Figure 6D; D, Representative images of TUNEL staining for Figure 6E; E, Representative images of immunohistochemistry for Figure 6F; F, Representative protein blots for Figure 6G; G, Protein blots for Figure 6H; H, Protein blots for Figure S1F.

**TABLE S1 | Primer sequences for reverse transcription quantitative polymerase chain reaction.**

| Gene | Primer sequences (5' - 3') |
| --- | --- |
| KLF4 | F: 5’-AGAGGAGCCCAAGCCAAAGA-3’  R: 5'-CAGTCACAGTGGTAAGGTTTCTC-3' |
| EZH2 | F: 5'-CGCCACCACTCACTACCACAC-3'  R: 5'-TGGATTTAATAGCGTGCTGCC-3' |
| GAPDH | F: 5'-CACGGCAAATTCAACGGCACAGTC-3'  R: 5'-ACCCGTTGGCTCCACCCTTCA-3' |

Note: F, forward; R, reverse; KLF4, Krüppel-like factor 4; EZH2, Enhancer of zeste homolog 2; GAPDH, glyceraldehyde-3-phosphate dehydrogenase.
